# Trajectories of Posttraumatic Stress Symptoms Among Young Adults Exposed to a Typhoon: A Three-Wave Longitudinal Study

**DOI:** 10.3389/ijph.2022.1605380

**Published:** 2023-01-04

**Authors:** Wei Shi, Brian J. Hall

**Affiliations:** ^1^ Institute for Disaster Management and Reconstruction (IDMR), Sichuan University, Chengdu, China; ^2^ Center for Global Health Equity, NYU Shanghai, Shanghai, China

**Keywords:** post-traumatic stress symptoms trajectories, resilience, recovery, deterioration, young adults, typhoon

## Abstract

**Objective:** We used a latent class growth model to identify distinct PTSS trajectories and correlates of these trajectories among young adults who experienced Typhoon Hato, the strongest storm to strike China in the last 50 years.

**Methods:** A longitudinal survey (three-waves) was conducted to explore the mental health status and its correlates among young adults exposed to the typhoon. Data from 362 participants were analyzed *via* a latent class growth model and multinomial logistic regression.

**Results:** Three distinct classes of PTSS trajectories were identified, including: “resilience” (86.46%), “recovery” (9.12%), and “deterioration” (4.42%). The higher levels of direct typhoon exposure, media use, and posttraumatic growth significantly predicted the higher likelihood of participants being in the “recovery’’ class. In addition, more social support significantly predicted the higher possibility of being in the “resilience” class. Finally, more severe depressive and anxiety symptoms significantly predicted the higher likelihood of being in the “deterioration” class.

**Conclusion:** Further research should develop interventions to enhance protective factors (e.g., posttraumatic growth, media use), decrease risk factors (e.g., depressive and anxiety symptoms), and thereby prevent PTSS.

## Introduction

Exposure to disasters (e.g., earthquakes, tsunamis, and hurricanes) can increase the risk of developing post-traumatic stress disorder (PTSD) [1]. PTSD is one of the most serious and common psychiatric disorders among people who are directly or indirectly exposed to traumatic events, and it includes symptoms such as intrusion, avoidance, numbing, and hyper-arousal [2]. Post-traumatic stress symptoms (PTSS) are commonly regarded as the negative psychopathological reactions following a traumatic event [2]. Untreated PTSS can lead to serious consequences and severe emotional dysfunction for people affected, including life-long chronic mental disorders and impaired functioning [1]. With growing threats of natural disasters worldwide [3], further research focusing on trajectories of PTSS is needed to develop a better understanding of cultural patterns of posttraumatic reactions that are currently underrepresented in the literature.

Previous studies have shown the deleterious consequences of PTSS followed by disasters. For example, a review analyzed 284 reports and found that the prevalence of PTSS ranged from 4.1% to 24.0% among survivors following various disasters [4], and another study demonstrated that 30.5% of Chinese adolescents showed PTSS after exposure to the Wenchuan earthquake [5]. A longitudinal study with a sample of 3,594 participants showed that 25.2% of respondents had PTSS after the 2011 Great East Japan Earthquake and Tsunami [6]. However, the majority of studies used a cross-sectional design, so the development trend of PTSS survivors exposed to disasters could not be dynamically tracked. Thus, studies have called for longitudinal research on this topic [6].

Traditionally, studies have relied on analyzing sample means or aggregated data to explore mental disorder trends among populations over time [7, 8]. In recent years, researchers have opted for person-centered approaches rather than variable-centered approaches to test the heterogeneity within populations *via* applying growth mixture modeling methods, such as latent class growth model (LCGM) analysis [9, 10]. LCGM analysis can be used to effectively identify distinct clusters of participants with similar psychological symptom trajectories and growth parameters [11].

Previous studies have reported various latent classes of PTSD symptom trajectories following traumatic events. For example, a previous study analyzed 885 Hurricane Katrina survivors and also detected three classes of PTSS, namely, moderate-decreasing (69.3%), high-decreasing (23.1%), and high-stable (7.6%) [12]. A prior study included 1,573 adolescent survivors of an earthquake and found five PTSD symptom trajectory classes, resistance (65.3%), relapsing/remitting (3.3%), recovery (20.0%), chronic dysfunction (7.2%), and delayed dysfunction (4.2%; Fan et al., 2015). Another study with a sample of 1,707 American youths after hurricane exposure found four PTSS trajectories: low-decreasing (34%), moderate-stable (33%), recovery (23%), and chronic (10%) [13]. Additionally, Sumner et al [14] also reported four trajectories of posttraumatic symptom dimensions (i.e., avoidance, re-experiencing, dysphoric arousal, anxious arousal, numbing, threat, and loss) among Hispanic or Latino traumatic harm survivors (N = 427), namely, chronic (13%–34%), recovery (17%–42%), delayed onset (15%–42%), and resilient (17%–37%) latent classes. Due to varied results in prior studies, more research using LCGM analysis should be conducted to further explore distinct PTSD symptom trajectories after a traumatic event. Based on the literature review, the following hypothesis (H1) was proposed:

H1: A three to five class model of PTSS trajectories will be identified.

Some risk factors significantly increase PTSS, such as demographics (being older, female and migrants), mental health status, exposure severity, and media use. For example, previous studies have reported that gender, age, and place of origin significantly influenced traumatic distress among survivors exposed to traumatic events [4, 10, 15]. Many studies have also indicated that depressive symptoms [6, 16], anxiety severity [17, 18], and stress symptoms [19, 20] were significantly associated with PTSS among disaster-exposed survivors. Moreover, researchers have reported that individuals with a more direct exposure to disaster events, such as typhoons [21], earthquakes [22, 23], and floods [24, 25], were more likely to suffer from PTSS. The General Conceptual Model of the Role of Stressors in the Etiology of Child and Adolescent Psychopathology suggests that direct exposure to stressful events could lead to psychological disorders *via* moderators, such as environmental contexts, individual characteristics, and mental health status [26]. Finally, negative or prolonged media use was found to be associated with higher PTSS during typhoons [27], hurricanes [28], and COVID-19 [29, 30]. The Conceptual Model of Mental Health Toll explains that excessive social media usage is significantly associated with severe traumatic distress during the COVID-19 outbreak [30]. Based on this research, the following hypotheses (H2, H3, H4, H5) were proposed:

H2: Demographics, including gender (being female), age (older age), and place of origin (Macao local) will be significantly associated with worsening PTSS trajectory class membership.

H3: Depressive, anxiety, and stress symptoms will be significantly associated with worsening PTSS trajectory class membership.

H4: Direct typhoon exposure will be significantly associated with worsening PTSS trajectory class membership.

H5: Media use will be significantly associated with worsening PTSS trajectory class membership.

Some protective factors have been reported to be helpful for PTSS prevention, including posttraumatic growth and social support. A previous meta-analysis of longitudinal studies analyzed 75 samples (32,402 participants) and evidenced that more social support was significantly associated with a decrease in PTSS [31]. In addition, a meta-analysis of 42 studies (11,469 participants) found a significantly positive or negative linear relationship between posttraumatic growth and PTSD symptoms [32]. Moreover, the Social Causation Theory proposes that a lack of social support resources can result in psychological distress [21, 22]. A series of theoretical models, such as the Conservation of Resources Model [33], the Stress-Buffering Model [34], and the Etiological Model of PTSD [31] suggest that social support could extend individuals’ mental wellbeing resources and buffer psychological stress. Furthermore, a longitudinal study followed 122 earthquake survivors and reported that posttraumatic growth was negatively associated with PTSS after a post-earthquake event [8]. The Action-Focused Growth Model proposes the close relationships between posttraumatic growth and traumatic experience. It situates posttraumatic growth as a form of “positive illusion” with an accommodative function for psychological distress adjustment, which could increase self-enhancement in the context of a traumatic threat [35]. Although many previous studies add to the understanding of related factors for PTSD outcomes, little is known about the influence of these key factors on distinct PTSS trajectories [9]. Based on research above, the following hypotheses (H6, H7) were proposed:

H6: Posttraumatic growth will be significantly associated with improving PTSS trajectory class membership.

H7: Social support will be significantly associated with improving PTSS trajectory class membership.

### The Current Study

In the current study, we aimed to examine the heterogeneity in a sample of young adults who experienced a serious disaster, Typhoon Hato. LCGM analysis was used to identify PTSS trajectories over the three-wave follow-up period. Moreover, we examined key influencing factors that were associated with PTSS trajectories, including demographic factors (e.g., age, gender, and place of origin), mental health status (depressive, anxiety, and stress symptoms), direct typhoon exposure, media use, social support, and posttraumatic growth.

## Methods

### Dataset

Typhoon Hato made landfall on 23 August 2017. It was the strongest storm to hit Macao in the last 50 years. Typhoon Hato led to more than 250 injuries, 10 deaths, and a financial loss of up to 1.42 billion dollars [27]. The data in the current study is from a longitudinal research program tracking mental health development among Chinese young adults exposed to Typhoon Hato [27, 36]. The participants were students recruited at the University of Macau, which was seriously damaged by Typhoon Hato. All participants experienced a lack of water, power, food, or medical supplies for at least 3 days in Macao.

Data were collected in three waves with 6-month time intervals: Time 1 (T1, 21 September–6 December 2017), Time 2 (T2, 3 April–3 May 2018), and Time 3 (T3, 3 September–3 October 2018). At T1, 1,921 valid responses were collected (66.1% women; Mean age = 20.0, SD = 2.68), at T2 965 were collected (71.6% women; Mean age = 20.8, SD = 2.80), and at T3 362 were collected (76.2% female; Mean age = 21.3, SD = 2.78). For the current study, we only included data from the 362 participants who finished all three study waves. No significant differences were found between the total student population and study population in demographics and research variables, except for gender, age, and place of origin (*p* < 0.05). Results showed that female, younger, Macao-local participants were more likely to be lost to follow up.

This longitudinal study was approved by the Research Ethics Committee of University of Macau. Online self-report questionnaires and attached informed consent were distributed to all potential participants *via* official university e-mails. Participants could tick the agreement option in the informed consent to reading the study purpose, privacy, participation risk, and data retention. A cash lottery prize of 100 Macau Patacas (around USD $13) was provided to 50 participants as a participation stimulus in each study wave.

### Measures

#### Posttraumatic Stress Symptoms

PTSS was evaluated by the Chinese Version of the PTSD Checklist for the DSM-5 (PLC-5) [37]. The PCL-5 includes 20 self-report items to determine PTSS over the past month [38]. Participants rated each item about Typhoon Hato *via* a five-point Likert scale (*0 = none, 4 = extreme;* e.g., “Have you had repeated, disturbing dreams of the typhoon Hato?”). A total score of the PCL-5 ranges from 0 to 80, and a higher score represents more severe PTSS [39]. Many prior studies have evidenced the good reliability (Cronbach’s α between 0.91 and 0.97) and validity in the Chinese version of the PCL-5 [40]. The data of PTSS was collected in each wave study. This scale also showed excellent reliability in each study wave (T1 Cronbach’s α = 0.97; T2 Cronbach’s α = 0.94; T3 Cronbach’s α = 0.95).

#### Depressive, Anxiety, and Stress Symptoms

Depressive, anxiety, and stress symptoms over the past week were measured by the Chinese version of the 21-item depression anxiety and stress scales (DASS-21) [41]. The DASS-21 consists of depressive, anxiety, and stress symptoms subscales, with 7 items in each. Each item is rated on a five-point Likert scale (ranging from 0 *= never, 3 = almost always*), and a higher score represents more severe symptoms. Sample items measuring depressive, anxiety, and stress symptoms are “I couldn’t seem to experience any positive feeling at all,” “I felt I was close to panic,” and “I found it hard to wind down,” respectively. The Chinese version of this scale has good reliability (Cronbach’s alpha is above 0.80 for the overall scale and above 0.90 for the subscales) [36, 40]. The data was collected at T1. In this study, the Cronbach’s alphas for the depressive, anxiety, and stress symptom subscales were 0.88, 0.84, and 0.88, respectively, indicating good reliability.

#### Direct Typhoon Exposure

Direct typhoon exposure was measured by 15 items using a dichotomous response (0 = no, 1 = yes). These items were developed from a meta-analysis that summarized risk factor exposure to previous natural disasters [42]. In this study, the items assessed the following: residence being damaged or flooded, injuries, death of loved ones, being trapped or stranded during the typhoon, witnessing traumatic events happening to others, or almost drowning in the flooding [36]. Items were summed to assess the extent of direct typhoon exposure, and a higher score represented more severe direct typhoon exposure. The data of direct typhoon exposure was collected at T1.

#### Media Use

Media use refers to the degree or number of uses in different social and transitional media platforms, such as television, radio, Facebook, and Wechat. Media use was assessed by 9 items focusing on the respondents’ direct exposure to media use during and 1 week after Typhoon Hato [27]. Eight questions tested the length of time spent using media for typhoon-related information, such as news, videos, and images *via* television, radio, the newspaper, and online platforms. A sample item was “How many hours did you spend listening to radio programs about Typhoon Hato?” (responses ranged from 0 h to >12 h). We rescored these eight questions using 0 = 0 h and 1=>1 h. One dichotomous question was used to assess the respondents’ sharing of typhoon-related information on personal media accounts. The sum of all items represented the extent of media use, and a higher score indicated more typhoon-related media use. The data of media use was collected at T1.

#### Social Support

Social support, or the perceived form of informational, emotional, physical, or practical assistance from families, friends, or significant people [43], was measured with the Chinese version of the 12-item Multidimensional Scale of Perceived Social Support [43]. Each item was rated on a seven-point Likert-type scale (*1 = very strongly disagree, 7 = very strongly agree;* e.g., “My family really tries to help me”), and higher scores indicated better perceived social support during the typhoon. Many previous studies have evidenced the good reliability of this scale with Cronbach’s alphas above 0.89 [36, 44]. The data of social support was collected at T1.The reliability of this scale was excellent in the current study (Cronbach’s α = 0.96).

#### Posttraumatic Growth

Posttraumatic growth, which is the discovery of benefits, thriving, and positive psychological changes after experiencing a traumatic event [3], was measured by the Chinese version of the Posttraumatic Growth Inventory (PTGI) [45]. The PTGI is the most commonly-used psychometric tool to examine posttraumatic growth after disaster events [3]. The PTGI includes 21 items rated on a 5-point Likert scale (*0= I did not experience this change as a result of my crisis, 5= I experienced this change to a very great degree as a result of my crisis*; e.g., “I changed my priorities about what is important in life”). The PTGI is scored by summing all responses. This scale has shown great reliability in previous studies (Cronbach’s α = 0.96) [46] and showed good reliability in the current study (Cronbach’s α = 0.97). The data of posttraumatic growth was collected at T1.

#### Demographics

Demographic information, such as age, gender, and place of origin were collected.

### Statistical Analysis

We used Mplus version 8.3 [47] and SPSS version 24.0 [48] to conduct data analysis in three stages. First, unconditional latent class growth analyses were conducted to explore the PTSD trajectory classes without covariates. The maximum-likelihood estimation was applied with missing data. Second, the descriptive analysis and correlation matrix were conducted for PTSD trajectory classes and study variables. An analysis of variance (ANOVA) or chi-square test were conducted to examine the significant mean differences among study variables between the PTSD trajectory classes. Finally, a multinomial logistic regression was performed to test the influencing factors significantly predicting the PTSS trajectory classes.

## Results

### Participant Characteristics

Table 1 shows the participants’ characteristics. This study included data from 362 Chinese young adults who completed all three study waves. Most participants came from Macao (*n* = 218, 60.22%), with the remaining participants being from outside of Macao (*n* = 144; 39.28%). Most participants were women (*n* = 276, 76.24%). Participants’ ages ranged from 18 to 36 (mean = 21.29, SD = 2.78). Furthermore, Time 1 participants provided data between 25 September and 24 November 2017. We conducted an analysis to determine differences in study populations across this period with regard to correlates and the outcome and found no statistical differences (*ps* > 0.001).

**TABLE 1:** Participants’ characteristics and identified posttraumatic stress symptoms trajectory classes (Macao, China, 2017–2018).

Variables	Total (N = 362)	Resilience PTSS class (*n* = 313; 86.46%)	Recovery PTSS class (*n* = 33; 9.12%)	Deterioration PTSS class (*n* = 16; 4.42%)	*p*
	M (SD) or n (%)	M (SD) or n (%)	M (SD) or n (%)	M (SD) or n (%)	
Sociodemographic characteristics					
Gender					
Male	86 (23.76%)	68 (21.73%)	11 (33.33%)	7 (43.75%)	
Female	276 (76.24%)	245 (78.27%)	22 (66.67%)	9 (56.25%)	
Age (18–36)	21.29 (2.78)	21.21 (2.77)	21.15 (1.87)	23.19 (3.859)	0.003
Place of origin					
Macao	218 (60.22%)	180 (57.51%)	24 (72.73%)	14 (87.50%)	
Non-Macao	144 (39.78%)	133 (42.49%)	9 (27.27%)	2 (12.50%)	
Mental Health Status					
T1 PTSS**	6.26 (10.40)	2.84 (3.63)	30.33 (9.71)	23.56 (17.01)	<0.001
T2 PTSS**	5.79 (8.49)	3.96 (5.66)	14.30 (12.25)	24.06 (12.71)	<0.001
T3 PTSS**	5.39 (8.80)	3.21 (4.41)	10.61 (7.42)	37.13 (10.40)	<0.001
Depressive Symptom	7.55 (7.77)	6.33 (6.75)	12.42 (8.12)	21.25 (8.91)	0.114
Anxiety Symptom	6.62 (6.95)	5.36 (5.53)	12.30 (8.22)	19.63 (9.83)	<0.001
Stress Symptom	8.73 (8.17)	7.447 (7.20)	15.58 (8.63)	19.75 (10.09)	0.062
Typhoon-related Exposure					
Direct Typhoon Exposure	2.69 (2.05)	2.49 (1.87)	4.00 (2.61)	3.75 (2.84)	0.002
Media use	6.77 (1.80)	6.67 (1.75)	7.45 (2.14)	7.31 (1.66)	0.259
Social Support and Personal Growth					
Social Support	5.29 (1.15)	5.40 (1.14)	4.55 (0.84)	4.64 (1.09)	0.109
Posttraumatic Growth	38.51 (23.65)	36.74 (23.38)	51.36 (20.60)	46.56 (26.22)	0.513

N: M, mean, SD, standard deviation; PTSS, posttraumatic stress symptoms; T = time (wave); *p*-value for Analysis of Variance (ANOVA) or Chi-square test.

### Intercorrelation and Multicollinearity Test

Table 2 shows the intercorrelations between independent variables. All independent variables except for depressive, anxiety, and stress symptoms showed small effect size associations with each other.

**TABLE 2:** Correlations coefficients between study independent variables (N = 362) (Macao, China, 2017–2018).

No.	Variables	1	2	3	4	5	6	7	8	9	10
1	Age	\									
2	Gender	−0.007	\								
3	Place of Origin	−0.071	−0.069	\							
4	Depressive Symptom	0.081	−0.071	0.190***	\						
5	Anxiety Symptom	0.079	−0.027	0.135*	0.744***	\					
6	Stress Symptom	0.086	−0.057	0.136**	0.757***	0.798***	\				
7	Social Support	−0.040	0.192***	−0.181***	−0.291***	-0.245***	−0.217***	\			
8	Direct Typhoon Exposure	−0.065	−0.048	−0.015	0.038	0.143**	0.101	−0.061	\		
9	Media use	0.037	0.056	0.105^*^	0.067	0.011	0.035	0.048	0.062	\	
10	Posttraumatic Growth	0.001	0.052	0.023	0.003	0.128*	0.082	0.221***	0.156**	0.236***	\

N: *p < 0.05, **p < 0 .01, ***p < 0.001; N_total_ = 362.

According to Miles [49], if the variance inflation factor (VIF) is higher than 4 and tolerance is smaller than 0.25, a serious multicollinearity problem can occur. The multicollinearity test results showed that VIFs (range: 1.03–3.34) and tolerances (range: 0.30–0.97) for independent variables were smaller than 4 and higher than 0.25, which suggested a low possibility of a multicollinearity between study variables.

### Posttraumatic Stress Syndrome Trajectory Classes

To discriminate trajectories for traumatic stress symptoms among young adults exposed to Typhoon Hato, we used the total PTSS scores to conduct an unconditional LCGM with intercept and slope parameters, comparing one to six class models. According to previous recommendations [50, 51], significant *p*-values of the bootstrap likelihood ratio test (BLRT) and Lo-Mendell Rubin likelihood ratio test (VLMR-LRT), a higher entropy coefficient, and lower Akaike information criterion (AIC), Bayesian information criterion (BIC), sample-size-adjusted Bayes information criterion (SABIC) scores indicated a better model fit. A *p*-values below .05 were considered significant. Table 3 shows the model fit indexes.

**TABLE 3:** Fit indices for latent class growth analysis examining posttraumatic stress symptoms across three study waves (Macao, China, 2017–2018).

Number of classes	AIC	BIC	SABIC	Entropy	VLMR-LRT (*p*)	BLRT (*p*)
1	7907.314	7926.772	7910.910	—	—	—
2	7476.588	7507.721	7482.341	0.964	0.5727	<0.001
3	7307.954	7350.762	7315.864	0.977	0.1516	<0.001
4	7215.017	7269.500	7225.085	0.962	0.4351	<0.001
5	7104.557	7170.715	7116.781	0.966	0.3363	<0.001

N: AIC, akaike information criterion; BIC, bayesian information criterion; SABIC, sample-size adjusted Bayesian information criterion; VLMR-LRT, Lo–Mendell–Rubin likelihood ratio test; BLRT, bootstrap likelihood ratio test; N_total_ = 362.

The 5- and 4-class models were excluded because of the small size of PTSS trajectory class (participants number in the class below 15) [52]. Results showed that the *p*-values of the VLMR-LRT were not significant in any of the class models. The *p*-value of the BLRT were significant in all class models, which has been suggested to be a stronger indicator of class model fit [53]. The AIC, BIC, and SABIC scores were smaller and entropy was higher in the 3-class model than the 2-class model. Consequently, the 3-class model was selected as the best model fit.

**FIGURE 1:**
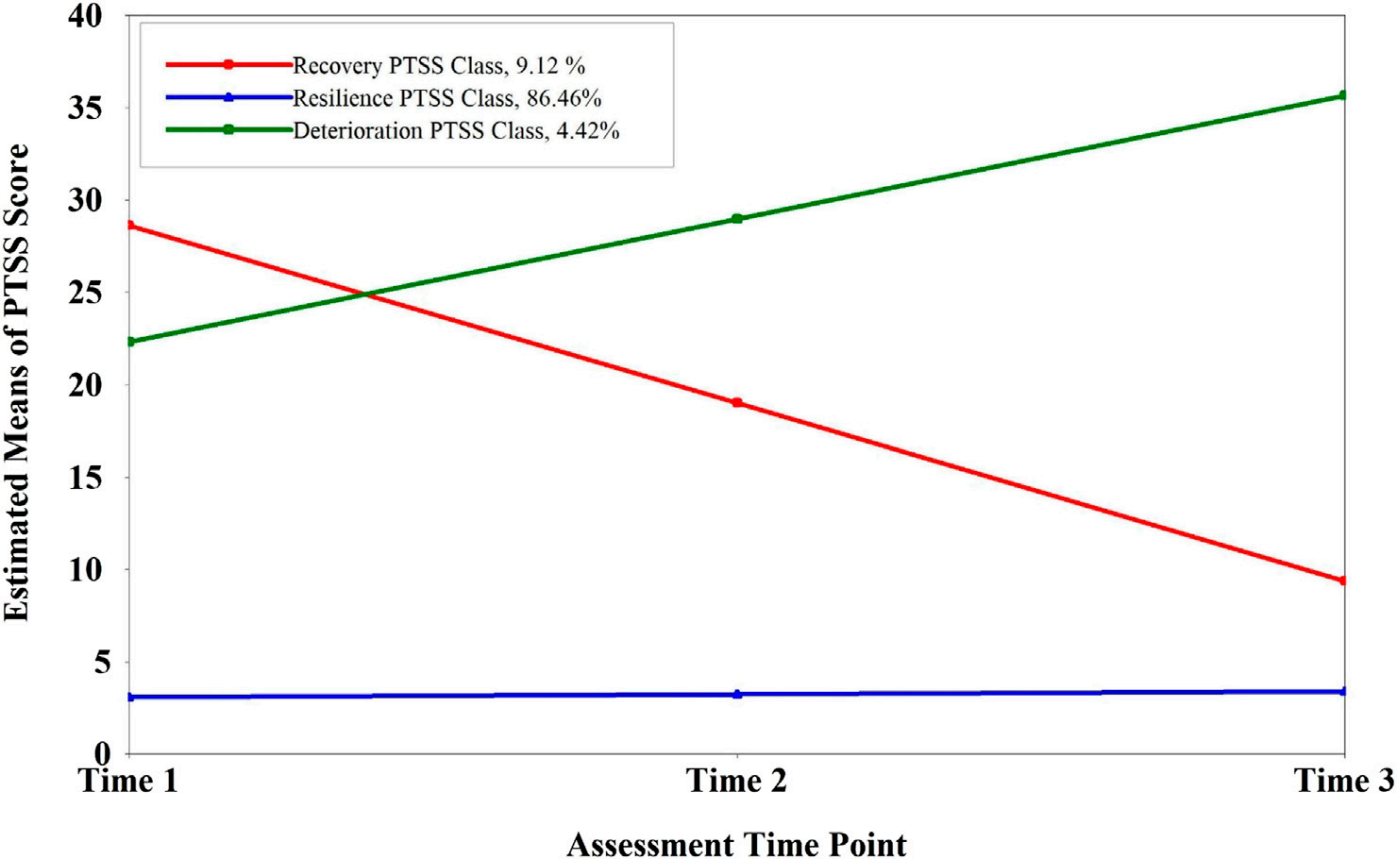
Longitudinal course of post-traumatic stress symptoms across three assessment time points as suggested by the unconditional 3-class model solution. (N = 362) (Macao, China, 2017–2018). PTSS, post-traumatic stress symptoms.

The 3-class model included three distinct trajectory classes of PTSS among the participants (see Figure 1). The first PTSS trajectory class, “resilience”, accounted for 86.46% (*n* = 313) of the sample and had consistently low and unchanged PTSS scores and a non-significant slope over time (*p* = 0 .328). The second PTSS trajectory class, “recovery” comprised 9.12% (*n* = 33) of the sample and had a consist reduction trend from relatively high PTSS scores and a significant slope parameter (*p* < 0.001). The third PTSS trajectory class, “deterioration,” contained 4.42% (*n* = 16) of the sample and had continuously ascending PTSS scores and no significant slope parameter (*p* = 0.087). Notably, the PTSS level of the “resilience” group was quite low when compared with the other two groups (i.e., “recovery” and “deterioration”) with high PTSS levels. These results supported H1 (see Table 4).

**TABLE 4:** Class-specific parameter estimates for the three-class solution (Macao, China, 2017–2018).

	Intercept				Slope			
PTSS trajectory classes	Est.	S.E.	Est./S.E.	*p*	Est.	S.E.	Est./S.E.	*p*
Resilience PTSS Class (N = 313, 86.46%)	3.08	0.24	13.02	<0.001	0.16	0.16	0.98	0.328
Recovery PTSS Class (N = 33, 9.12%)	28.63	3.62	7.91	<0.001	−9.63	2.31	−4.18	<0.001
Deterioration PTSS Class (N = 16, 4.42%)	22.30	5.34	4.18	<0.001	6.68	3.90	1.71	0.087

N: N_total_ = 362; Est. = parameter estimates; S.E., standard error; PTSS, posttraumatic stress symptoms.

### Multinomial Logistic Regression Results

Table 5 presents the results of the multinomial logistic regression, which explored the significant predictors for the three PTSS trajectory classes. The “resilient” class was the more frequently occurring class and it was used as the reference category in logistic models. The “resilience” PTSS trajectory class was selected as the reference class for the regression models. If the relative risk ratio (RRR) was higher than 1.0, the predictive association indicated a higher likelihood of falling into the latent class compared with the reference class, and *vice versa*. Based on the results of the likelihood ratio (LR) chi-square test, a model containing the full set of predictors presented a significant improvement in fit relative to a null model, LR χ^2^(20) = 135.63, *p* < 0.001, Pseudo *R* ^2^ = 0.39. The full model including all predictors represented a 38.87% improvement in fit relative to the null model.

**TABLE 5:** Multinomial logistic regression analysis of posttraumatic stress symptoms outcome (Macao, China, 2017–2018).

	Recovery & resilience PTSS classes				Deterioration VS resilience PTSS classes			
Predictors	B	S.E.	*p*	RRR	B	S.E.	*p*	RRR
Age	−0.02	0.08	0.793	0.98	0.18	0.09	0.057	1.20
Gender								
Men	Ref	\	\	\	Ref	\	\	\
Women	−0.42	0.49	0.390	0.65	−0.96	0.74	0.196	0.38
Place of origin								
Non-Macao	Ref.	\	\	\	Ref.	\	\	\
Macao	0.43	0.48	0.367	1.54	1.43	0.97	0.139	4.18
Depressive Symptom	0.01	0.04	0.859	1.01	0.16	0.06	0.007	1.17
Anxiety Symptom	0.06	0.05	0.223	1.06	0.20	0.07	0.004	1.22
Stress Symptom	0.06	0.04	0.106	1.07	-0.10	0.07	0.156	0.90
Social Support	-0.75	0.20	<0.001	0.47	-0.33	0.33	0.324	0.72
Direct Typhoon Exposure	0.23	0.09	0.012	1.25	0.22	0.15	0.157	1.25
Media use	0.32	0.14	0.022	1.38	0.38	0.25	0.133	1.46
Posttraumatic Growth	0.04	0.01	0.001	1.04	0.02	0.02	0.266	1.02

N: N_total_ = 362; PTSS, posttraumatic stress symptoms; RRR, relative risk ratio; Ref. = Reference group.

We found that social support was a negative and significant predictor of being in the “recovery” class (B = −0.75, S.E. = 0.20, *p* < 0.001, RRR = 0.47). For every one unit increase in social support, the log-odds of an individual falling into the “recovery” PTSS trajectory class was significantly predicted to decrease by 0.75 units. Moreover, for every one unit increase in social support, the relative risk of being in the “recovery” PTSS trajectory class (relative to the risk of being in the “resilience” PTSS trajectory class) changed by a factor of 0.47. The results partially supported H7.

The results showed that direct typhoon exposure (B = 0.23, S.E. = 0.09, *p* < 0.05, RRR = 1.25), media use (B = 0.32, S.E. = 0.14, *p* < 0.05, RRR = 1.38), and posttraumatic growth (B = 0.04, S.E. = 0.01, *p* < 0.01, RRR = 1.04) were positive and significant predictors of individuals being in the “recovery” class. For each unit of increase in these three predictors, the log-odds of an individual falling into the “recovery” PTSS trajectory class were significantly predicted to increase by 0.23, 0.32, and 0.04, respectively. Moreover, for each unit of increase in direct typhoon exposure, media use, and posttraumatic growth, the relative risk of being in the “recovery” PTSS trajectory class (relative to the risk of being in the “resilience” PTSS trajectory class) changed by the factors of 1.25, 1.38, and 1.04, respectively. These results rejected H4 and H5 but supported H6.

We found that depressive (B = 0.16, S.E. = 0.06, *p* < 0.01, RRR = 1.17) and anxiety (B = 0.20, S.E. = 0.07, *p* < 0.01, RRR = 1.22) symptoms were positive and significant predictors of individuals being in the “deterioration” PTSS trajectory class. For each unit of increase in level of depressive and anxiety symptoms, the log-odds of an individual falling into the “deterioration” PTSS trajectory class were significantly predicted to increase by 0.16 and 0.20, respectively. Moreover, for each unit of increase in level of depressive and anxiety symptoms, the relative risk of being in the “deterioration” trajectory class (relative to the risk of being in the “resilience” PTSS trajectory class) changed by factors of 1.17 and 1.22, respectively. In addition, we found that stress symptoms and demographics (i.e., gender, age, and place of origin) were not significant predictors of any PTSS trajectory classes. These results partially supported H3 and led us to reject H2.

## Discussion

As we hypothesized that three- to five-class PTSS trajectories could be identified (H1), the current three-wave longitudinal study investigated three distinct PTSS trajectory classes (i.e., resilience, recovery, and deterioration) *via* LCGM analysis. Several significantly positive (i.e., depressive symptoms, anxiety symptoms, direct typhoon exposure, media use, and posttraumatic growth) and negative (i.e., social support) predictors of PTSS trajectory classes were found. To our knowledge, this is the first three-wave longitudinal study to identify distinct PTSS trajectory classes and their influencing factors among young adults exposed to a disastrous typhoon in Macao. The results can provide a possible explanatory mechanism for associations between long-term heterogeneous PTSS trajectories and key influencing factors after disasters.

We identified a three-class PTSS trajectory, which is different from the trajectory classes found in prior studies with different populations [13, 14, 54]. Previous studies have commonly reported a relatively common “resilience”, “recovery,” and “chronic” PTSS trajectory classes [10]. Consistent with previous research, the largest group (86.46%) was the “resilience” PTSS trajectory class, followed by the “recovery” PTSS trajectory classes (9.12%) in the current study. This suggests that most survivors who were retained in this longitudinal study handled exposure to the disaster reasonably well and did not need psychosocial intervention within the year after experiencing the massive Typhoon Hato. Additionally, it should be noted that some survivors could have a delayed emotional reaction to traumatic events, which may change the “resilience” into “delayed” PTSS trajectory classes, namely showing a consistent low risk of PTSS at several beginning timepoints but followed by an increase in PTSS in later timepoints [10, 53]. Moreover, there was a small group in the “deterioration” PTSS trajectory, which indicates a need for professional interventions to mitigate PTSS. Based on these results, future studies should conduct longer-term research to further explore PTSS trajectories and related psychotherapy [25].

The results showed that participants receiving higher social support were more likely to be in the “resilience” PTSS trajectory class rather than the “recovery” PTSS trajectory class. This result partially supported H7 that social support would significantly link with improving PTSS trajectory class. This suggests that social support could be a shielding factor for PTSS but not a key factor diminishing PTSS among young adults after a disaster. This result was different from previous studies, which reported that social support was significantly correlated with decreased PTSS symptoms [54, 55]. Some previous studies found that higher social support was associated with decreased risks of experiencing traumatic distress [31, 56]. One possible explanation for these different results is that when individuals experience child abuse, neglect, apathy, and family dysfunction, they find it difficult to perceive the support around them [57, 58]. In addition, childhood adversity and trauma can result in difficulties with intimacy, sociability, and help-seeking for support among adolescents [59, 60]. Thus, among people with negative childhood experiences, social support may not directly affect reduced PTSS. Thus, future studies should examine the mechanism of association between social support and PTSS *via* a mediator or moderator, such as childhood experience, traumatic history, and family mutuality [31].

Another notable finding was that higher levels of direct typhoon exposure and media use significantly predicted a higher probability of membership in the “recovery” PTSS trajectory class. This result did not support the H4 and H5 that direct typhoon exposure and media use could significantly associate with worsening PTSS trajectory class. These results were inconsistent with some existing research, which reported that more traumatic exposure and media exposure could lead to more severe PTSS [23, 29, 30]. However, several prior findings could provide explanations for the different results. For example, studies have found that individuals can develop positive traits during trauma exposure, including constructive stress management skills, and positive self-belief [61] or receive optimistic information *via* media [27], which could assist in PTSD prevention. Another study found that increased optimism, gratitude, and recovery during trauma exposure were significantly associated with PTSD mitigation [61]. Hall et al [27] found that exposure to more information about the storm itself and seeing images of heroic behaviors significantly predicted a lower risk of PTSD symptoms. In contrast, previous studies have also shown that direct trauma exposure [21, 23, 62] and prolonged media use could increase PTSD symptoms [30, 63, 64]. Thus, as suggested by Hall et al [27] and Shi et al [65], future studies should explore the role of positive personal traits (e.g., appreciation and recovery) during disaster exposure and positive information obtained through social media (e.g., heroic pictures and stories) on mental wellbeing after exposure to disaster events.

We found that a higher level of posttraumatic growth significantly predicted a higher risk of being in the “recovery” PTSS trajectory class. This result is consistent with H6 that posttraumatic growth could significantly link with improving PTSS trajectory class. Some previous findings that more posttraumatic growth can benefit PTSD prevention [8, 32]. Moreover, previous studies have explained possible paths to traumatic events eliciting posttraumatic growth [66]. Cognitive processing could be stimulated by posttraumatic stress, and when it is converted into constructive processing, people can renovate the schema and assumption, leading to positive alteration in cognition and reduced distress [8]. In recent years, researchers have shifted focus from negative outcomes to positive psychological changes, including posttraumatic growth, following traumatic events. Future studies can further examine the positive changes after traumatic events, such as the changes in social relationships, spiritual appreciation, and life philosophy [8].

We found that higher depressive and anxiety symptoms, except stress symptoms among participants, significantly predicted the increased risk of being in the “deterioration” PTSS trajectory class. These results partially kept in line with H3 that depressive, anxiety, and stress symptoms would significantly associate with exacerbating PTSS trajectory class. Previous research has consistently supported the positive relationships of depression and anxiety with PTSD [16, 17]. Although depression and anxiety could raise the risk of suffering from PTSD [67], PTSD could also contribute to greater depressive and anxiety symptoms following traumatic events [68]. Several overlapping and common risk factors could exist independently among depression, anxiety, and PTSD [69, 70]. Thus, future studies should explore the mechanisms of comorbid PTSD with depressive and anxiety symptoms [69]. Moreover, we found demographics, including gender, age, and origin of place did not present a significant influence on the PTSS trajectory, which rejected H2. The results suggested these personal inherent characteristics could not be crucial factors in predicting PTSS trajectory. Future studies need to further focus on the influencing mechanism of some external factors, (e.g., social support, mental health status, and social media use) on PTSS trajectories.

### Limitations

There were several limitations in the current study. First, we used three waves of data obtained during the year after Typhoon Hato, however, PTSS trajectories could be different in longer wave studies because of possible delayed traumatic symptoms among survivors exposed to a disaster. Longer-term studies should therefore be conducted to further track the PTSS trajectories among young adults after a disaster. Second, the sample size was relatively small, as the large loss to follow-up reduced the sample size and may have biased the current results. Thus, future longitudinal studies should incorporate novel retention strategies. Third, the current study only tested common risk or protective factors influencing PTSS trajectories because of the limited questionnaire. Future studies should include other possible factors that may affect PTSS trajectories, such as family psychiatric history and personality [21]. Finally, some variables, such as social support and posttraumatic growth were only measured at Time 1. Future studies should continuously track their effects and changes alongside the PTSS presentation over time, as they may be time-varying.

### Conclusion

The current study used a novel person-centered approach to identify heterogeneous trajectories of PTSS among young adults after Typhoon Hato: “resilience,” “recovery,” and “deterioration” PTSS trajectory classes. Moreover, some significant protective factors were found to decrease the risk of PTSS, including direct typhoon exposure, media use, and posttraumatic growth. Some risk factors significantly influenced increasing PTSS, such as depressive and anxiety symptoms. These results have potential clinical implications, such as identifying individuals in “resilience” and “recovery” classes, who may not need costly intensive intervention service after a traumatic event. In terms of practical implications, a program focusing on promoting posttraumatic growth and improving social support from family or peers should be developed, which could protect disaster-exposed survivors from PTSS after a disaster. In addition, a more targeted professional PTSS intervention may be needed for individuals in the “deterioration” PTSS trajectory class. More evidence is needed on characteristics of membership in the “deterioration” class that may help decrease the risk of falling into this“deterioration” PTSS trajectory class. Future investigations should conduct a longer-term study to explore more protective factors influencing PTSS trajectories, which could help elucidate the mechanisms of recovery and improve related interventions for adults after a disaster.
